# Round the Hospitals

**Published:** 1920-07-17

**Authors:** 


### ROUND THE HOSPITALS.

It is announced from the Ministry of Health that Miss Marian Scott Riddell, R.R.C., has been appointed by the General Nursing Council to be the Registrar of the Council, and that Dr. Addison has sanctioned the appointment. Miss Riddell was trained at St. Bartholomew's Hospital and was formerly matron of the Chelsea Hospital for Women. During the war she was Matron of the 2nd London General Hospital, and subsequently of the 53rd General Hospital in France, until she was appointed Principal Matron, and afterwards Acting Matron-in-Chief of the Territorial Force Nursing Service. It is a good augury for the future of the Nursing Council that they have been able to secure the services of this distinguished woman in helping to reorganise the nursing profession.

**ROUND THE HOSPITALS—(*continued*).**

The new number of the *College Bulletin*, which carries the report of the elections and the annual meeting to all members, contains an important and very clearly worded announcement of the College position in respect to registration. It is quite true that in the early stages of propaganda for State registration a Bill was drafted by the College in which it was provided that, in the event of the College Bill becoming an Act of Parliament, all the nurses on the College Register would automatically become State-registered nurses without further fee, the College at the same time handing over its funds to the General Nursing Council and becoming thereby absorbed by it. This Bill did not become law, and probably all the College nurses are now very glad that it did not, because it is abundantly clear that there is a wide sphere of work outside registration for the College to perform. The College authorities now announce their willingness to pay the registration fee to the extent of a guinea for any nurse who may have joined the College before March 18, 1920, under the belief that the College undertook to pay the registration fee for its members, whatever registration Bill might ultimately become law. We have no expectation that this claim will be made by many nurses. The desire is all the other way with College members. They are already dissatisfied with the small amount they have paid for the privileges accorded them, and are casting about how best they may supplement the finances of their great society.

---

A garden fête was held, by permission of the King, in the grounds of St. James's Palace on July 9, at which the Earl of Athlone, Princess Alice Countess of Athlone, the Duchess of Albany, and Princess Marie Louise received the workers of the League of Mercy. The Prince of Wales is Honorary Grand President, and the King is Patron of the League, which, as our readers know, was founded by King Edward VII., on the inspiration of the late Sir Henry Burdett, for the purpose of inviting and collecting small subscriptions to the hospitals. In the course of twenty-one years it has fulfilled its purpose to the extent of £293,000 paid in through the King's Fund to London hospitals and convalescent institutions. The gardens were a blaze of decorations, and many members had travelled great distances in order to be present. Tea was served in the garden and in the Palace, where the Earl of Athlone, Princess Alice, and Princess Marie Louise presided at tables for eight guests. The band of the 2nd Life Guards played throughout the afternoon, and the scene was animated.

---

While the King and Queen were inspecting the Soldiers' Home of the Royal Scots Greys at Retford on July 9, Miss Davidson, foundress of St. Columba's Hospital, formerly known as Friedenheim, was presented to their Majesties. The Queen is Patron of St. Columba's, and remembers being present at its opening by the Duchess of Teck thirty-three years ago. Miss Davidson celebrated her eightieth birthday a short time back, on which occasion a message of hearty good-will and greetings was sent to her from the hospital, where the annual meeting was in progress. Colonel W. McAdam Eccles presided on this occasion, and gave an interesting account of the work done in this "home for those who are leaving the earthly tabernacle of their body." It has always been distinguished by the very beautiful sense of invisible forces which dominates and sustains alike medical officers, nurses, and patients, shedding an air of cheerfulness and serenity over the whole establishment. The present matron is Miss McNeill, and Colonel Eccles expressed to her and "to the splendid staff who work with and under her, not forgetting the cook," the thanks of all connected with the hospital.

---

The Queen performed a congenial task on Saturday in visiting the Edinburgh School of Cookery and Domestic Economy in Atholl Crescent, for Her Majesty's housewifery instincts render her a warm supporter of all that makes for efficiency in the sphere of home. The students ventured upon an offering of their handiwork in the form of shortbread and chocolates to the Queen and Princess Mary, which was graciously received. The activities of the school extend beyond what is usually included in domestic science courses, and the Queen expressed much admiration when she was informed that the rooms were painted and papered and the furniture upholstered by women students, while she inquired with a smile, "And what is the attitude of the trades unions to this?".

---

The Archdeacon of Hampstead officiated, on July 2, at the unveiling of a bronze memorial tablet by Alderman Wooley Walden, Chairman of the M.A.B. Hospitals Committee, in the hall of the Nurses' Home at Colindale Hospital, Hendon, to the memory of three brave nursing sisters who were drowned during the war on hospital ships. Julia Winchester was drowned on the *Falaba*, March 21, 1915; Mary Rodwell on the *Anglia*, November 7, 1915; and Isabella Cruickshank on the *Solta*, April 10, 1917. The tablet was subscribed for by members and friends of the Central London Sick Asylum Nurses' League, to which the above nurses belonged, having been trained in the establishment now known as the Colindale Hospital. This fine institution is now entering upon a new sphere of work. The matron, Miss Smith, recently recovered from a severe illness, was present at the unveiling and received many congratulations on her return to active work.

---

Miss S. Sharpe, matron of Atkinson Morley Convalescent Hospital, Wimbledon, which is the convalescent branch of St. George's Hospital, has resigned after thirty years' service as matron, and is retiring in October on a pension. She will be succeeded in the matronship by Miss Edith A. Watkins, the present matron of the General Lying-in Hospital, Lambeth. Miss Watkins was trained at St. George's Hospital, and was sister there for several years.

**ROUND THE HOSPITALS—(*continued*).**

THE inquiry by the Bermondsey Board of Guardians into the alleged complaints as to the administrative staff at their infirmary, referred to in THE HOSPITAL on June 19, page 308, ended, as was generally expected, in the complete vindication of the nursing and administrative staff. A letter, to which was attached the initials of twenty patients, stating that they had been threatened by a staff nurse, led to a series of explanations and apologies all round, with the result that all were satisfied, and it is hoped that the work will go on smoothly in the future. The Committee reiterated their opinion that the bed-clothing was sufficient, that the food was plentiful and well cooked, and that there was no reason at the infirmary for the unusual number of resignations of the nursing staff except that they were over-worked, due to the shortage of nurses. They decided that in future when a nurse resigns she shall be interviewed by the Committee. Another good proposal was that the medical superintendent, Dr. Harkness, should report on his proposal for the formation of a Consultative Committee for the staff. This suggestion was somewhat novel, but there is no reason why a Committee should not be formed, and lead to staff grievances being removed more quickly than they are at present.

CONSIDERABLE ferment has been excited at the Kingston Infirmary by the recent incident in connection with the visit of some of the nursing staff to the Derby. It was reported to the Guardians that Miss Sinclair, the matron, had made use of the infirmary ambulance on June 3, Derby Day, in order to visit the racecourse, and she was invited to be present at a meeting of the Guardians to explain her action. Unfortunately, Miss Sinclair did not attend the meeting or offer any explanation, and the Guardians have asked for her resignation. Miss Sinclair was interviewed by a representative of the *Evening Standard* on July 7, and stated that she was fagged out at the time, and, knowing that the ambulance would not be used till 2.30, she and three of her nurses, craving for fresh air, drove down to Epsom in it. She sent the driver back in the middle of the day, and the ambulance fetched the party in the evening. She subsequently went to Scotland, where she spent a fortnight of her leave in bed, and, returning to attend the meeting of the Guardians, to which she had been summoned, found herself too ill to appear before them. With every inclination to make allowances for a craving for fresh air, the Guardians could hardly pass over this breach of professional etiquette, nor can we think it is quite fair to complain that Miss Sinclair was condemned without a hearing.

THE London County Council school nurses seem to have been moving through troubled waters lately to judge by a resolution moved at the London Teachers' Association by Mr. Shortt, of Lewisham, making it a punishable offence for parents to use violent or abusive language to teachers on the school premises. It appears that the new cleansing scheme is not working smoothly, and that mothers whose children are "superficially clean" are indignant when the school nurse orders something more to be done. It is an invidious duty, and both teachers and nurses are entitled to protection in carrying it out.

WHAT was described as "open rebellion" on the part of the nursing staff at the Gelligaer Council's isolation hospital, South Wales, against the doctor's orders was reported at the Council's meeting last week. The matron and two of the nurses wrote tendering their resignations, the former saying it was advisable she should state the reasons for her drastic step, which were that after five months' working with the medical officer, Dr. J. Thomas, she found she could not expect anything like harmony to exist, or hope to keep her charge nurses. Dr. Thomas, who was present, informed the Council that an impossible situation had arisen at the hospital during the past few days. Owing to his great anxiety with regard to certain children at the hospital, he had to give orders, he said, for certain treatment to be administered, but he afterwards found that his instructions had not been carried out. It became, he added, a question of open rebellion, as the matron and sisters refused to submit to his orders, and he could not accept the responsibility for the welfare of children at the hospital if his lines of treatment were not carried out. It was at first proposed that the Council should accept the resignations of the matron and nurses, but eventually an amendment was adopted referring the trouble to the Hospital Committee for investigation, all the parties involved to be invited to attend. Thereupon Dr. Thomas declared that he looked upon the Council's decision as practically a vote of censure upon him, and, declining to attend any inquiry, he placed his resignation as medical superintendent in the Council's hands.

NURSING in South Africa should receive a great impetus from the coming of Princess Arthur of Connaught as Vicereine. While every member of our Royal Family has shown an interest in the sick and suffering, Princess Arthur has in a peculiar manner fitted herself to speak with authority on nursing and nurses. For two years she worked in the wards of St. Mary's Hospital, Paddington, and, except that she did not sleep in the hospital, shared in all other respects the routine of the nurses' life. Towards the end of her time she was allowed to assist in the theatre, a privilege of which both she herself and others of the family were exceedingly proud. It was a testimonial to her efficiency. After St. Mary's the Princess went to Queen Charlotte's Hospital, and was trained in maternity nursing, so she has considerable experience to draw on. At St. Mary's she helped in the nursing of soldiers, and there she made determined and successful efforts to overcome the shyness which the gallant men felt at being cared for by a member of the Royal Family. On one occasion she told the patients that she noted that they said

**ROUND THE HOSPITALS**—(*continued*).

"Good morning" to all their other nurses, but never to her. For a while they were tongue-tied, but at last a bold Canadian managed to say "Good morning, Nurse Connaught," and the Princess's pleasure at the greeting encouraged the rest of the ward to do the same. Her Royal Highness claimed no privileges and was a strict disciplinarian. It is said that when Princess Maud called one morning, her sister refused to let her come into the ward, saying, "You can't come in now, these are not visiting hours." The nursing world will look forward to great developments in South African nursing when Princess Arthur of Connaught goes to Capetown.

Hospitals are at last waking up to a perception that a large source of revenue lies ready to hand almost untouched in patients' payments. The Great Northern Central has always had paying wards, and they have been much in request by middle-class patients. But from now on every in-patient, unless really incapable of contributing, will be expected to pay part of the expense of treatment. The charge for cubicle wards will be from £1 1s. to £4 4s., and in the private wards from £4 4s. to £6 6s. In the general wards terms will be fixed to suit the means of the patients, and will range from about 5s. to £1 1s. a week. Very few persons are incapable of paying as much as 10s. to £1 a week for all the benefits received in hospital, and their combined support will relieve the charity of an enormous strain. Some of the older hospitals are prohibited by the terms of their charter from adopting this necessary measure, and it has become a matter for earnest consideration whether application should not be made for such alterations as would enable them to march with the times.

The women students at Oxford have adopted an academic dress of unobtrusive appearance, with a distinctive square-shaped hat. We are wondering what steps the colleges will take if it becomes the fashion for typists, nursery governesses, and shop assistants to wear the same uniform and pose as students. If means have been adopted to prevent the abuse of the collegiate garments it would be interesting to the nursing profession to know how this has been managed. Nurses have suffered, and do suffer, severely from a too flattering imitation of their garb by women unqualified to use it, who (this is the crux of the uniform problem) do not always behave in character when they assume the outward appearance of the nurse. We anticipate that someone will do a good business in "colourable imitations" of the academic costume.

The East Sussex County Nursing Federation shows in its admirable report that its secretaries (Lady Mabelle Egerton and Mrs. W. F. Foster) fully appreciate the importance of the work to which they have laid their hands, and have a wide grasp of nursing organisation. Miss Scott, Matron of the Sussex County Hospital, testifies to there being a marked decrease in the number of ill-nourished babies admitted to the hospital and brought to the out-patient department, and this is one striking way in which the long patient labours of nurses and midwives may be brought to public notice. The committee declare in their report that the heart of the sound organisation which has been developed lies in the village. Certainly Sussex, so far as our own observation goes, owes a debt of gratitude to the zealous and devoted village nurse midwives who make up a large proportion of its nursing service

Among the many events of last week was a picturesque garden fête and French market, opened by Prince and Princess Arthur of Connaught, in the grounds of Chiswick House, in aid of the West London Hospital, of which Prince Arthur is President. There were most attractive stalls of flowers, fruit, vegetables, and dairy produce, and the open-air dancing was a very pretty feature, somewhat spoiled by the uncertainty of the weather.

Miss Charlotte Aikins has done a real public service in opening up the condition of the hospitals in South America through her series of articles, to which we have before alluded, in the *Trained Nurse*, New York. She urges upon her readers in the June number that the need for nurses and health workers cannot be overestimated, but cautions intending pioneers not to attempt to go out to South America without a mastery of conversational Spanish or (should Brazil be the spot selected) Portuguese. The lack of any species of night nursing is a scandal which reflects the gravest discredit on the States responsible. In a great many hospitals we learn there is not even an orderly in wards containing from forty to fifty beds. The doors are locked at night, and the sick get on as best they can. One nurse employed on the West Coast stated that it was not unusual to see five or six bodies carried out of one of these wards in the morning before work began. Operative patients apparently doing well "slipped away" unattended at night.

Two important hospitals in Detroit have decided to shorten their course of nurse-training to two and a-half years for ordinary probationers and two years for collegiate women. It is thought probable that this retrograde step will be followed throughout the State of Michigan. Detroit has been suffering from a great shortage of candidates, and the authorities are evidently not of opinion that a high nursing standard is an attraction to students at the training-schools. The Michigan hospitals have recently held a publicity campaign for candidates, which has had the desired effect of filling all the classes. But we are persuaded the authorities are making a fatal mistake in encouraging the desire for hasty training which no doubt exists in the lay world in America and in this country.

